# La historia contra la amnesia: la epidemia de influenza en América Latina

**DOI:** 10.1590/S0104-59702025000100040

**Published:** 2025-09-15

**Authors:** Marcos Cueto

**Affiliations:** i Editor cientifico, investigador, Casa de Oswaldo Cruz/Fiocruz. Rio de Janeiro – RJ – Brasil cuemarcos@gmail.com

Esta valiosa compilación de 11 capítulos examina una pandemia, erróneamente conocida como “Gripe Española”, que entre 1918 y 1920 se propagó rápidamente por todo el planeta, causando entre 36 y 40 millones de muertes – más víctimas que las de la Primera Guerra Mundial, que terminó en 1919. A pesar de su extensión y envergadura, no ha recibido la atención adecuada por parte de los historiadores con notables excepciones como los de María Isabel Porras Gallo, de España, y Claudio Bertolli Filho, de Brasil (Bertolli Filho, 2003; Porras Gallo, Davis, 2014). Otras excepciones a esta falta de atención son autores presentes en algunos de los capítulos, investigadores que ya han publicado trabajos relevantes sobre el tema (Bertucci, 2005; Carbonetti, 2021; Molina del Villar, Márquez Morfín, 2022). Este libro (Altez, Molina del Villar, Arrioja Díaz Viruell, 2023) cumple con dos objetivos de los historiadores de la salud: primero confirma que una epidemia actúa como un lente de aumento para comprender procesos históricos, y, en segundo lugar, expone con profunda claridad cómo las condiciones de vida de la población magnifican la enfermedad generalizada.

Los capítulos, resultado de reuniones de una red especializada en la historia de las epidemias, no se limitan a describir y analizar lo ocurrido en Argentina, Bolivia, Brasil, Chile, Colombia, Costa Rica, Guatemala, Honduras, México y Venezuela. También abordan las diversas vías de entrada y el recorrido de la pandemia en cada país, el impacto desigual en las capitales y provincias, y la interacción entre las respuestas oficiales y el estigma social.

Aunque el libro dedica atención a los factores cualitativos, como el contexto previo a la epidemia, el pánico colectivo y la propaganda de tratamientos – la mayoría ineficaces, como vacunas, quinina y eucalipto, dado que en ese entonces no se sabía mucho de la dolencia –, el énfasis recae en los aspectos cuantitativos y descriptivos, que se presentan mediante detallados cálculos, mapas, gráficos y tablas. Además, la obra incluye un detallado índice toponímico, algo útil que no es común en los libros de historia publicados en español.

La obra se caracteriza por un exhaustivo trabajo de investigación que abarca una amplia variedad de fuentes primarias y secundarias, tales como informes oficiales, artículos periodísticos, documentos de archivo y, en ciertos casos, actas de defunción, lo que refleja una inmersión en los materiales disponibles para reconstruir los eventos de la pandemia.

De acuerdo con una tabla presentada al inicio del libro, los países que registraron el mayor número de fallecimientos fueron México y Brasil, que, en conjunto, sumaron cerca de medio millón de muertes, una cifra altísima para una epidemia (Brasil tenía entonces alrededor de 30 millones de habitantes y México unos 14 millones). Una de las principales fortalezas de la obra radica en reconocer sus propias limitaciones, como la imposibilidad de abarcar todos los países de la región o la naturaleza incompleta de las fuentes disponibles sobre la epidemia. Los datos sobre mortalidad, los más confiables, presentan deficiencias debido a posibles subregistros, mientras que las estimaciones relacionadas con la morbilidad (el número de personas que enfermaron) y la letalidad (la proporción de muertes entre los enfermos) resultan particularmente complejas y difíciles de determinar.

Los especialistas en esta pandemia podrán contrastar lo ocurrido en Europa y América del Norte con las de la región a partir de este texto. Entre las similitudes destaca el hecho de que, en casi todas partes, los grupos más afectados fueron los adultos jóvenes, especialmente aquellos entre los 20 y 30 años, un segmento etario generalmente resistente a las enfermedades respiratorias, ya que estas solían afectar a niños y ancianos. Hubo una cierta variabilidad en cuanto a las tres olas de la pandemia que se sucedieron entre 1918 y 1920. La mayoría de los países experimentaron una mayor letalidad en la segunda ola, a finales de 1918, aunque algunos solo sufrieron los efectos devastadores de la última ola, la de 1920.

Un rasgo de la región fue la forma en que la pandemia llegó y se expandió a través de diversos medios. En la mayoría de los países, el contagio ocurrió por medio de barcos de inmigrantes provenientes de Europa, así como a través del comercio marítimo de empresas como la United Fruit Company, que tenía una presencia hegemónica en el Caribe y Centroamérica. En Brasil, el único país de la región que participó en la Primera Guerra Mundial, la influenza se propagó, además de las embarcaciones procedentes de Europa, con soldados que regresaban de las trincheras europeas donde la dolencia era endémica. En México, un punto de contacto crucial fue la frontera norte con los EEUU. No obstante, en todas partes, la influenza se diseminó gracias a la red de ferrocarriles de reciente construcción.

Una característica en América Latina fue la participación de curanderos y médicos “charlatanes”, tanto hombres como mujeres, quienes se sumaron a las discusiones sobre los tratamientos más efectivos, disputando con las autoridades y los médicos graduados en universidades la hegemonía sobre las respuestas sanitarias.

Dos críticas a este libro son que, a pesar de tener una excelente introducción que sitúa el problema general, muchos trabajos empiezan repitiendo un resumen de la pandemia. Asimismo, no existe un capítulo final que sistematice las similitudes y diferencias con las formas en que se manifestó la pandemia en otras regiones del mundo. No obstante, estos problemas son comunes en muchos libros editados, y los colaboradores de este volumen deben ser disculpados, ya que han logrado realizar un avance significativo en el conocimiento.

De particular interés son tres temas clave que atraviesan todos los capítulos. El primero es que, en desastres sanitarios, el Estado adquiere una dimensión preponderante y autoritaria, aunque siempre marcada por inestabilidad, respuestas tardías y una capacidad limitada para mitigar las dolencias. El segundo tema son los detalles de cómo la historia fragmentada de las instituciones de salud y las precarias condiciones sociales de vida potencian la vulnerabilidad de los pobres. Finalmente, en tercer lugar, la tendencia, quizás nunca tan marcada como en esta pandemia, de las autoridades y la sociedad a olvidar estos desastres una vez que han cesado sus estragos.

¿Por qué? Creo que ni los autores, ni el autor de esta reseña, tenemos una respuesta clara para comprender esta amnesia, pero me atrevo a proponer que el cortoplacismo que rige la vida de los gobiernos y las personas les impide vislumbrar el largo plazo, ya sea hacia el pasado o hacia el futuro. En todo caso, estos temas son fundamentales para comprender cómo las calamidades de salud, el autoritarismo y la amnesia se retroalimentan, resultando en que las epidemias se repitan cíclicamente. Un ciclo pernicioso y destructivo al que los historiadores de este libro han hecho frente sugiriendo que la principal lección de la historia es que necesitamos de más investigación histórica.
